# Theories of schizophrenia: a genetic-inflammatory-vascular synthesis

**DOI:** 10.1186/1471-2350-6-7

**Published:** 2005-02-11

**Authors:** Daniel R Hanson, Irving I Gottesman

**Affiliations:** 1 Department of Psychiatry, VA Medical Center (116A), One Veterans Drive, Minneapolis, MN, 55417 and Departments of Psychiatry & Psychology, University of Minnesota, USA; 2 Departments of Psychiatry & Psychology, University of Minnesota, Minneapolis, MN 55454, USA

## Abstract

**Background:**

Schizophrenia, a relatively common psychiatric syndrome, affects virtually all brain functions yet has eluded explanation for more than 100 years. Whether by developmental and/or degenerative processes, abnormalities of neurons and their synaptic connections have been the recent focus of attention. However, our inability to fathom the pathophysiology of schizophrenia forces us to challenge our theoretical models and beliefs. A search for a more satisfying model to explain aspects of schizophrenia uncovers clues pointing to genetically mediated CNS microvascular inflammatory disease.

**Discussion:**

A vascular component to a theory of schizophrenia posits that the physiologic abnormalities leading to illness involve disruption of the exquisitely precise regulation of the delivery of energy and oxygen required for normal brain function. The theory further proposes that abnormalities of CNS metabolism arise because genetically modulated inflammatory reactions damage the microvascular system of the brain in reaction to environmental agents, including infections, hypoxia, and physical trauma. Damage may accumulate with repeated exposure to triggering agents resulting in exacerbation and deterioration, or healing with their removal.

There are clear examples of genetic polymorphisms in inflammatory regulators leading to exaggerated inflammatory responses. There is also ample evidence that inflammatory vascular disease of the brain can lead to psychosis, often waxing and waning, and exhibiting a fluctuating course, as seen in schizophrenia. Disturbances of CNS blood flow have repeatedly been observed in people with schizophrenia using old and new technologies. To account for the myriad of behavioral and other curious findings in schizophrenia such as minor physical anomalies, or reported decreased rates of rheumatoid arthritis and highly visible nail fold capillaries, we would have to evoke a process that is systemic such as the vascular and immune/inflammatory systems.

**Summary:**

A vascular-inflammatory theory of schizophrenia brings together environmental and genetic factors in a way that can explain the diversity of symptoms and outcomes observed. If these ideas are confirmed, they would lead in new directions for treatments or preventions by avoiding inducers of inflammation or by way of inflammatory modulating agents, thus preventing exaggerated inflammation and consequent triggering of a psychotic episode in genetically predisposed persons.

## Background

When the solution to a clinical or scientific puzzle eludes us for more than a century, as with schizophrenia (formerly dementia praecox), we need new ways of thinking about the problem [1,2]. Efforts to understand schizophrenia have focused on neurons and, especially, the role of presumed excess dopamine neurotransmission. We believe that genetic, environmental, and stochastic factors combine with epigenetic factors to create episodes of the illness [3-5]. Thus, the syndrome of schizophrenia is viewed as an endpoint in a dynamic *process *variously conceptualized as degenerative or developmental or alternating at different points in the process [6-10].

Degenerative models imply that after a period of normal development, the organism, or one of its parts, takes a wrongful turn in its trajectory and begins to malfunction. This describes the eventual outcome for all life forms and is a biological restatement of the second law of thermodynamics. Since degeneration is universal, stating that an illness is degenerative is not particularly helpful. What would be helpful is to determine when in the life course the degeneration begins and how the degeneration is initiated and proceeds. Answers to the "when?" and "how?" questions would then describe the *degenerative *process in *developmental *terms.

Developmental models of schizophrenia implicate abnormalities of early brain development predisposing to future schizophrenia. The proponents of the model further argue that the perturbations of development are limited to the early times of development and are discontinuous. Without this qualifier, developmental models are indistinguishable from degenerative models where the degeneration commences early in the life span. The early abnormalities are not necessarily the cause of schizophrenia, but, instead, create a state of risk for a future episode of schizophrenia. That is, a diathesis or predisposition is not a disease. Consequently, there must be factors later in life that convert the vulnerability to an illness. These additional factors are presumed to damage development in such a way that a predisposition becomes actualized. To gain a complete understanding of the syndrome, we must again return to the question of " what happens?"

Following this line of reasoning, the distinction between degenerative and developmental models blurs. In fact, a medical-behavioral condition can be both developmental and degenerative as exemplified by Down syndrome [11-13]. Individuals born with trisomy 21 exhibit a number of developmental anomalies including cardiac malformations, abnormal dermatoglyphics, skeletal changes, and muscular hypotonia, to name a few. As trisomy 21 infants mature, most exhibit degrees of mental retardation. By about age 50, these individuals invariably develop Alzheimer-like CNS degenerative changes that can be seen at autopsy [13].

Schizophrenia involves both developmental and degenerative features. From the time of Bleuler [14] and Kraepelin[15], "It is certain that many a schizophrenia can be traced back into the early years of the patient's lives..." [14] p. 252. The 'follow back' studies of schizophrenia support these views [16]. Likewise, prospective studies of children at high risk for schizophrenia report developmental anomalies in motor skills, cognition, and attention long before the onset of overt illness [17-19]. Overt psychotic symptoms for some individuals usually start in the late teenage years or early twenties, but the illness can start as early as middle childhood [20] and may, more rarely, start in old age [21] p 73].

The evidence suggesting early developmental perturbations in schizophrenia is compelling. At the same time, there certainly are examples of deterioration reminiscent of Kraepelin's suggestion for some people with schizophrenia. However, deterioration in clinical course may not indicate CNS deterioration. Instead, the decline could be a secondary consequence of an illness that disrupts education, economic achievement, and social functioning leading to a downward spiral in all aspects of adult life. Consistent with an early degenerative process, there are reports of declining cognitive function preceding onset of psychosis [22]. Proponents of neurodevelopmental models suggest that the premorbid cognitive abnormalities are developmental risk factors for future schizophrenia (c.f [23]) and argue that such abnormalities show little evidence of decline after onset [6,24]. Whether developmental or degenerative, the premorbid cognitive deficits seen in schizophrenia are also seen in other disorders [25] and lack specificity and sensitivity thus detracting from the concept that the cognitive abnormalities seen in schizophrenia are useful endophenotypes [26]. The strongest evidence for a neurodegenerative phenomenon comes from imaging studies showing progressive loss of brain volumes [27-29]. Neuropathological studies fail to find widespread classic signs of neurodegeneration such as gliosis though there are exceptions to this generalization [30]. Observations of abnormal dendritic arborization [31,32] are consistent with the neuroimaging evidence suggesting abnormal connectivity between brain regions [29]. As a cautionary note, most of the neuroimaging and neuropathology results are subject to confounds from the effects of medications and various other treatments, post-mortem intervals, possible effects of diet, smoking habits, as well as a myriad of other potential confounds associated with glucocorticoid mediated stress following chronic illness and associated life's limitations [33,34].

The symptoms of schizophrenia are highly variable. Within families (and thus presuming relative homogeneity of genetic and environmental factors) symptoms can vary widely over time, as illustrated by identical quadruplets concordant for schizophrenia [35]. Even within affected individuals, symptoms will wax and wane and may even remit [36] suggesting a life long process.

The major behavioral symptoms of schizophrenia include alterations in cognition, memory, perception, thought (inferred from language), motor functions, and affect. People with schizophrenia may show abnormal dermatoglyphics and other minor physical anomalies [37-42]. Other oddities to be incorporated in a comprehensive explanation of schizophrenia include highly visible nail fold capillaries [43,44] and the rarity of rheumatoid arthritis among schizophrenic persons [45]. These physical characteristics suggest the need to look beyond the nervous system *per se *to have a comprehensive view of the illness.

The fact that the schizophrenia syndrome, as currently defined, is relatively common provides important information about the frequency of causal factors. About 1% of the population will experience schizophrenia during the lifespan. Except for a few rare exceptions, this 1% risk is remarkably constant around the globe regardless of culture, geography, or ethnicity. Men and women are affected equally. These facts mean that the risk factors for schizophrenia must also be common and ubiquitous. Given that the concordance rate for schizophrenia in identical twins [46] is only about 50%, there must be at least two global risk-increasing categories for schizophrenia, i.e., something(s) genetic and something(s) environmental. Assuming these risk factors are independent of each other, the joint probability of acquiring both risk factors is the product of their population frequencies that, for schizophrenia, equals about .01. To make a simplifying assumption to allow easy calculations, let us say that the two risk factors are present with about equal frequency in the population. With this simplification, straightforward mathematics indicates that the individual frequencies of these factors are close to the square root of the population frequency of 1%. That would mean that about 10% of the population would encounter at least one risk factor. The math indicates that the greater the number of independent risk factors, the more common they are. [See [47] for further elaboration].

Our challenge is to develop a theory of schizophrenia that can plausibly explain an illness that affects all domains of behavior (thought, affect, motor performance, etc), that has elements of developmental perturbations early in life leaving clues such as minor physical abnormalities, and also has elements of degenerative changes. At the same time, the defect is so subtle that we can't find the cause(s) with our best modern technology. Furthermore, in spite of brain-wide dysfunctions, many individuals with schizophrenia remain sufficiently intact that, with good treatment and a bit of luck, can maintain jobs and function usefully in society. Thus, we need to find frequent and ubiquitous factors that can affect virtually all brain functions as well as creating somatic signs, but they operate in ways that leave these functions only slightly "off kilter" as compared to the complete disruption seen in strokes, or classical degenerative disorders such as Alzheimer, or as seen in Down syndrome where the behavioral pathology is apparent from earliest stages. As we try to explain schizophrenia, we must account for most all of the developmental and degenerative features of schizophrenia.

To account for the panoply of signs and symptoms seen in schizophrenia, any complete theory of schizophrenia must include organism wide systems. In addition to the nervous system, the immune system and the vascular system are defensible candidates. Both are invoked in the following theory: Some schizophrenia psychoses are the result of damage to the micro-vascular system in the brain initiated by genetically influenced abnormal inflammatory processes acting in response to ubiquitous environmental factors that trigger inflammatory responses, including infection, trauma, or hypoxia. It is the relative infrequency of the vulnerable genotypes in the population [48] that results in only a small proportion developing overt psychosis.

We wish to emphasize that our hypothesis specifically identifies the microvascular system as the critical site of inflammation. We postulate that the inflamed micro-vessels lose their coupling with astrocytes, leading to disrupted regulation of cerebral blood flow and damage to the blood brain barrier. These disruptions in homeostatic mechanisms then lead to abnormal signal processing. Our focus on inflammation of the vessels differentiates our hypothesis from models of widespread parenchymal inflammation such as seen in psychotic syndromes following, for example, encephalitis lethargica, or paraneoplastic syndromes. Many acute inflammatory disorders of the brain involve inflammation of both the parenchyma and the vasculature. By contrast, we are proposing a chronic, smoldering, inflammation of the vessels alone. And, finally, we distinguish our hypothesis from the theories of schizophrenia implicating direct parenchymal infection of the brain (c.f. [49]) and also differentiates our hypothesis from speculations about schizophrenia that invoke infectious agents altering DNA [50].

Many prior debates about inflammation in the brains of people with schizophrenia have focused on the presence of absence of gliosis (see [51] for review). The consensus opinion is that gliosis, though present in some cases, is not a consistent feature of the neuropathology of schizophrenia. However, as Harrison [51] points out, evaluating gliosis is fraught with a multitude of problems and is not a definitive indicator of degenerative/inflammatory changes in the brain. More recent efforts have demonstrated activation of microglia in the brains of some individuals with schizophrenia implying an ongoing immunopathological process in addition to what ever happened early in development [52]. Ongoing neurodegenerative processes are suggested by increased levels of S100B, a small calcium binding astrocytic protein that is involved in inducing apoptosis and modulating proinflammatory cytokines [53-55].

It is likely that the current clinical syndrome of schizophrenia is etiologically heterogeneous. We do not pretend to explain all (DSM or ICD) cases of syndromal schizophrenia. Instead, we put forward our hypothesis as an attempt to define a psychiatric syndrome in terms of a particular pathophysiology. Following this course may then help refine our nosology (see also section on 'specificity' below) and cause us to recalculate basics 'facts' such as prevalence rates.

## Discussion

### A primer on CNS blood supply

Neurons derive their energy from oxygen and glucose delivered by the vascular system, plus lactate and glycogen derived from astroglia [56]. The combination of neurons, astroglia, and micro-vessels form a metabolic trio [56] whereby the glia extend processes interacting with neurons on the one hand and, on the other, form endplates interdigitated into capillary walls. Rather than being passive conduits, the CNS vascular system is the most precisely managed and the most complex fluid dynamic system known. Regulation of cerebral blood flow (CBF) is managed primarily by a coupling between astrocytic glial cells [56-59] and capillary endothelium [60-65]. Astrocytes sense local neuronal metabolic activity and adjust blood flow as needed. Cerebral vessels change caliber in response to vasoactive substances released by astrocytes activated by glutamate receptors [56,66,67]. Serotonin [68], acetylcholine [69] and dopamine [66,70,71] transmission between astrocytes and micro vessels also play roles. When the neuronal activation of discrete areas is sustained over longer periods, vasoactive substances stimulate angiogenesis resulting in increased capillary density [67] thus enhancing local neuronal circuitry. Conversely, decrease in capillary density is likely to reduce the functional capacity of brain areas so affected [67]. Consequently, capillary beds in the cortex are not distributed in uniform fashion [72]. There are close relationships among local neuronal activity, density of capillary bed, and the distribution of valve-like flow control structures [73].

Developmentally, the CNS vascular system originates from capillary endothelial cells that migrate into developing neuro-ectoderm under the influence of trophic factors such as vascular endothelial growth factor (VEGF) [74] and erythropoietin [75] both produced by astroglia. The developing micro-vasculature, although comprising only 0.1% of the entire brain, and operating under the influence of genetic directives, has a key role in the development, maintenance and repair of the brain [76]. In turn, VEGF has trophic effects on neurons and glial cells, and the activity of VEGF influenced angiogenesis is directly proportional to the high metabolic activity of neocortical development [77]. Thus, angiogenesis and neurogenesis occur simultaneously and synergistically [78-80]. In addition to formation of capillaries themselves, intricate anastomoses between micro-vessels further 'fine tune' the metabolic support of developing glia and neurons [81]

### The genetics of infectious & inflammatory diseases

When infectious agents give rise to inflammatory vascular disease, the nature of the infectious agent may be less important that an individual's genetically influenced inflammatory response. The concept that infectious disease may have a genetic component is, of course, not new. Many agricultural geneticists make their livings by breeding disease resistance into both plants and animals [82,83]. One of the founders of behavioral genetics, Franz Kallmann [84], showed genetic factors influenced acquiring tuberculosis (DZ concordance = 26%, MZ concordance = 87%), an observation that was confirmed in modern times [85,86]. Many other infectious diseases appear to have genetic factors influencing susceptibility or resistance to the infection [87-97]. Mechanisms for genetically mediated responses to infection occur through genetic variations in immune mediators such as cytokines[96] and HLA factors [98,99].

Familial Mediterranean Fever (FMF) [100,101] provides a heuristic Mendelian example. The gene for FMF is located on the short arm of chromosome 16 and produces pyrin (marenostrin) that functions in a negative feed back loop to suppress inflammation. Absence of pyrin leads to exaggerated inflammatory responses. Vasculitis is one of the consequences [102]. Additionally, very high rates of rheumatic fever (RF) or rheumatic heart disease (RHD) are found in relatives of patients with FMF[103]. Having even one mutant gene appears to lead to immune hyperactivity to streptococcal antigens. We also know that antibody [104] production and cytokine activity [105] in RF patients is more marked than non-rheumatics. It is clear that genes influence the host's response to infection. A similar line of reasoning applies to other inducers of inflammation such as traumatic injury [106] or hypoxia [107,108].

Just as the CNS blood supply is highly regulated, the inflammatory systems in the brain require 'fine tuning.' Given the limited ability for adult brain to regenerate, and assuming there is little tissue to spare, it would make sense that the brain should be protected from overabundant inflammatory reactions [109]. Astrocytes play a key role in the expression of inflammatory cytokines, chemokines, and growth factors involving the modulation of gene expression for these factors [109-111].

Let us suppose that schizophrenia develops following an infection (or trauma or anoxia – the environmental contributors) but the host's response is determined by genetic factors regulating the nature and degree of inflammation. That infectious agents may be operative in schizophrenia is supported by several of lines of evidence. Summaries can be found in numerous sources [49,50,112-116]. The same concept applies to trauma [106] or anoxia [79,107] that may also stimulate inflammatory processes.

### Vascular disease and psychopathology

The syndrome of schizophrenia is likely to be etiologically heterogeneous and a multitude of CNS disorders can give rise to schizophrenic-like psychoses [117]. The idea that CNS micro-vascular diseases, in particular, are factors in psychotic disorders is also an old idea [118,119] that deserves a second look in light of new perspectives offered by developments in the genetics of inflammatory diseases. There are many examples of psychoses resulting from micro-vascular CNS disease including lupus and Sjögren syndrome [120]. Neuroimaging and neurocognitive deficits in these disorders are similar to those seen in schizophrenia [121]. Psychoses associated with substance abuse are also associated with CNS vasculitis [122]. Furthermore, infectious agents such as syphilis [123] and rheumatic fever (RF – see below), lead to micro-vascular disorders of the CNS that are associated with psychiatric symptoms including psychoses. Thomas, et. al. [124] also demonstrated small vessel abnormalities in the depressed elderly. At the same time, there is growing interest in cytokines and other inflammatory agents in psychoses[125] as well as growing awareness that inflammatory reactions are modulated by neuropeptides [126].

Inflammatory processes often damage the precise regulation of cerebral blood flow. The wide spectrum of clinical conditions thought to be created, in part, by inflammatory CNS micro-vessel disease include Alzheimer disease where it is thought that inflammatory processed damage the micro-vascular endothelium causing insufficient blood flow leading to oxidative stress, a build up of amyloid, and eventual cell death [127-135]. Cerebral palsy is also conceptualized as an infectious-inflammatory-vascular disorder where the vascular lesion is complete thrombosis [136]. Neurotoxic effects of methamphetamine and cocaine appear to be due to induction of inflammatory genes in small vessel endothelial cells [122,137], thus explaining the vascular damage seen in amphetamine and cocaine abuse that was previously attributed to contaminants of injected drugs [122,138-140].

Returning to the early stages of life, we have seen that the development of the neurons and glia are intimately associated with, and dependent on, the parallel development of the CNS vasculature. If the stated theory is correct, and given the developmental perspective of schizophrenia ---early developmental perturbations of the CNS set the stage for later schizophrenia--- we would expect to find support for the idea that inflammatory events early in life affect CNS vascular function. Such is the case. Whether the early insults are traumatic, infectious, or hypoxic; inflammatory process are involved in the attempts to protect and repair by modulating angiogenesis [141-148]. Thus, the reports implicating pregnancy and birth complications (anoxia, trauma or maternal infections) in the development of some cases of schizophrenia [149,150] could all be mediated by the common pathway of inflammatory-vascular mechanisms. Individuals who's genes created perturbations in inflammatory-vascular regulation would continue to experience abnormalities of protection and repair in response to subsequent CNS insults. Over time, the accumulation of 'hits' could lead to brain dysfunction to the extent seen in psychoses. The greater the number and duration of 'hits,' the greater the risk for a deteriorating /degenerative course. That neuroleptics may alter the permeability of the blood brain barrier and modify immunoregulation in the CNS [151] strengthens the argument for early treatment as a strategy to prevent deterioration.

### Alterations of cerebral blood flow in schizophrenia

Since the time of Seymour Kety's pioneering efforts [152,153], there has been interest in altered cerebral blood flow in people with schizophrenia. An in-depth review of this large literature is beyond the scope of this paper. The interested reader is referred to discussions of reduced anterior cerebral perfusion leading to the concept of 'hypofrontality' in schizophrenia [154,155] and to more recent reviews [156-158]. Bachneff's [159] review and theory about defects in regulation of CNS microvascular systems is particularly relevant. These reviews summarize a consistent body of evidence showing reduced cerebral blood flow in brains of people with schizophrenia especially to anterior regions. Flow deficits are seen in medication-naive new onset cases [160,161] and more established cases free of neuroleptics [162] suggesting that flow perturbations are neither the consequence of duration of illness nor treatment. Neuroleptics can alter cerebral blood flow [163,164] although the effects may be regionally and drug specific [165,166]. Decreased frontal flow is often associated with negative symptoms [167,168]. In addition to the frontal cortex, flow abnormalities in people with schizophrenia have been noted in the cingulate cortex [169,170], thalamus [171], basal ganglia [172], parietal cortex [167,170] and cerebellum [171]. Furthermore, in some instances, flow rates are increased [160,170]. Rather than a simple hypothesis of hypofrontality in schizophrenia, theorizing is evolving toward a concept of "dysfunctional circuits"[160] or "inefficient dynamic modulation" [173] of cerebral metabolism which is supported by other examples of abnormal modulation of cerebral blood flow in response to activation tasks [171,174]. Disturbances of blood flow in schizophrenia are well documented but are not limited to schizophrenia. Disturbed cerebral blood flow is also reported in obsessive compulsive disorder [175] and depression [176,177] as well as in Alzheimer disease (cited earlier). The usual interpretation is that alterations of blood flow arise as a consequence of abnormal neuronal metabolism. The theory proposed by this paper turns the causal arrow around to suggest that abnormalities of blood flow lead to altered neuronal-glial function that, in turn, leads to psychopathology. There has been scant direct visualization of the vascular system in schizophrenia, but at least one laboratory has found evidence of atypically simplified angioarchitecture and failure of normal arborization of small vessels [32].

### Post- streptococcal behavioral syndromes as a model

Post-streptococcal neuropsychiatric syndromes include Syndenham chorea, the PANDAS/obsessive compulsive syndrome, tics including Tourette syndrome, and possibly, ADHD [178-184]. Psychotic disorders are also implicated [183,185] and see citations below.

Sydenham chorea is the best-known neuropsychiatric complication following streptococcal pharyngitis. The association of psychoses and Sydenham chorea as well as with RF even in the absence of chorea, was discussed in the 17^th ^and 18^th ^centuries starting with Sydenham himself (see [186]). The interest in psychoses associated with RF continued throughout the 1900's [187-197]. People with a history of Sydenham chorea and/or rheumatic fever are at high risk for developing psychopathology later in life [198,199] with a relative risk for schizophrenia as high as 8.9 in a 10 year follow-up of 29 Sydenham patients [200]. There is a suggestion that the family members of Sydenham patients are also at higher risk for psychosis [201].

During the 1940's-1960's when RF was still quite prevalent, people with psychoses appeared to have higher than expected rates of histories of RHD or RF)[195,202,203] or rheumatic chorea [204]. Psychotic patients with RHD more often had early (<age 19) onset, movement disorders, progressively insidious courses and poor long-term outcomes [203]. Preliminary data from a Minnesota study also finds increased rates of RHD in psychotic patients, a pattern of increased psychiatric hospitalization following an epidemic of RF, and a clinical course for "rheumatic psychoses" that disproportionately led to a severe and continuous decline in function [205]. Although schizophrenia-like psychoses were the most common psychopathology related to rheumatic syndromes, manic-depressive, involutional, and senile psychoses were also observed [183,197].

An inflammatory reaction of the CNS vascular endothelium (vasculitis) is a common denominator in the both acute and chronic cerebral consequences of rheumatic fever. [186,187,190,195,197,206-209]. The microvascular lesions suggest both an obliterating process likely due to micro-emboli from rheumatic cardiac valves and an inflammatory process involving irregular proliferative changes in the vascular endothelium, dilatation of the lymphatic spaces surrounding the capillaries suggesting increased permeability of the capillary endothelium, and inflammatory cell infiltrates. Disruption of the blood brain barrier suggested by the evidence of increased permeability of the small vessels could compromise the immunological protection of the brain leading to the formation of the anti-neuronal antibodies seen in post-streptococcal CNS syndromes. In parallel fashion, people with schizophrenia show evidence of altered blood brain barrier and consequent alterations in immunological markers [210]

The post-strep psychopathologies provide a precedent for the hypothesis of this paper by demonstrating that an infectious process can trigger a series of inflammatory reactions that lead to a variety of somatic and psychiatric syndromes, including psychoses where vascular pathology is implicated. The pathogenicity of a strep infection is a function of the strain (genotype) of the bacterium and the genetically mediated inflammatory mechanisms of the host [211] and illustrates how a ubiquitous and often relatively benign environmental factor can create more serious sequelae in a limited number of genetically predisposed individuals-true genotype by environment interaction.

## Summary

**Figure 1:**
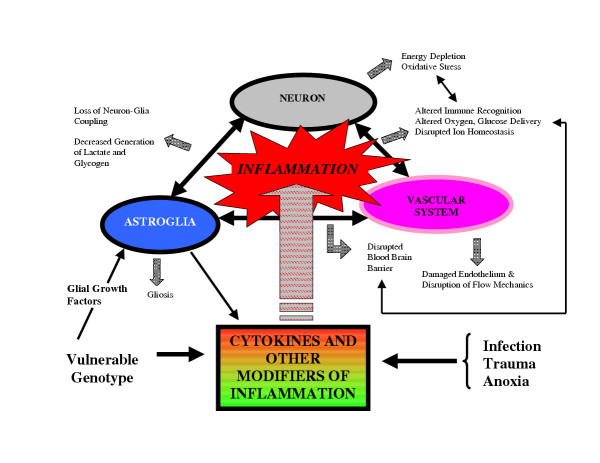
Simplified schematic illustrating the interconnected vascular-glial-neuron triad and how inflammatory processes may disrupt normal function.

The ideas here are not completely new. Eugen Bleuler [14] remarked: "The fragility of the blood vessels which appears in many schizophrenics, both acute and chronic, seems to indicate a real vascular pathology (p.167)." We bring old ideas forward into the light of new understandings offered by molecular genetics and inflammatory diseases. Since the late 1800's there has been evidence of inflammatory neuro-vascular abnormalities in psychiatric illness that were initiated by infectious agents. CNS lues (syphilis) is the best-known example. This paper expands the concept to suggest that a variety of environmental insults (infection, trauma, anoxia) may follow a common final pathway to psychopathology by stimulating inflammatory processes that damage the capillary-glial-neuron triad as illustrated in Figure 1.

Abnormal behaviors develop as a result of disruptions in astroglial mediated coupling of cerebral blood flow to neuronal metabolic needs. These subtle disruptions are hard to find, as the microvasculature comprises only about 0.1% of the brain and are of a scale more appropriate for electron microscopy. None-the-less, the hemodynamic perturbations have sufficient impact to cause subtle but widespread disruption of the normally harmonious coordination of CNS function leading to a condition variously conceived as a "neurointegrative defect"[212], "synaptic slippage" [213], "abnormal signal transduction" [4], "inefficient dynamic modulation" [173] or "synaptic destabilization" [214]. The ultimate impact would lead to psychopathology including psychoses as the vascular-glial-neuron triad is progressively damaged over time after repeated inflammatory episodes. The resultant failure to regulate the delivery of oxygen and energy adequately would lead to oxidative stress [215-217]. Oxidative stress, in turn, can further damage the microvasculature and the blood brain barrier [218-220]. The astroglial-capillary partnership that protects the integrity of the blood brain barrier would be compromised, thus exposing neural tissue to damage from immunological attack [221]. Known precedents of such processes are found in the behavioral changes seen in CNS vascular inflammatory diseases such as lupus and the post-strep syndromes described above.

**Figure 2:**
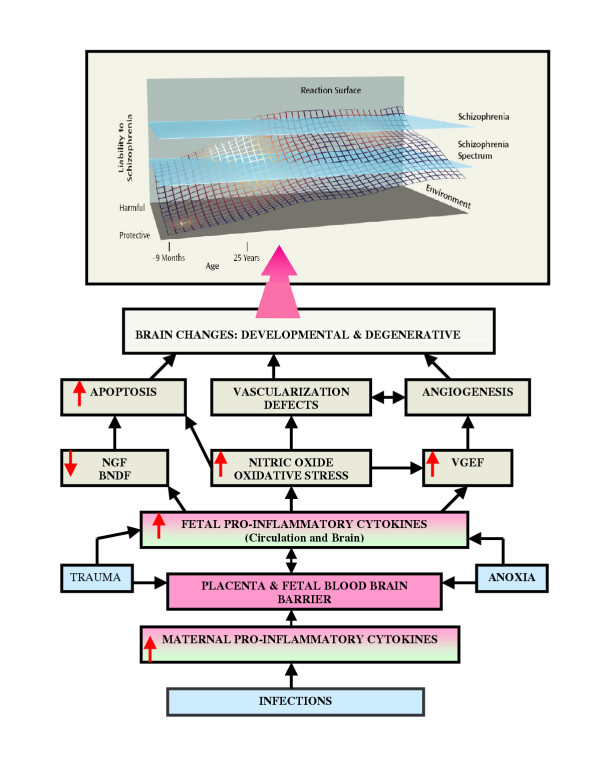
Schematic illustration of how inflammatory processes, from conception onward, may lead to CNS damage or dysfunction that dynamically alters the epigenetic landscape (reaction surface) thus affecting the liability for developing schizophrenia. Blue planes intersecting the reaction surface indicate levels of liability above which symptoms become manifest. Measurable factors in the middle of the figure are good candidates for endophenotypes. Adapted from [261,262].

This theory could explain how developmental events such as prenatal infections [150,222], and other birth and pregnancy complications [149] including anoxia [223] are linked to later schizophrenia – infection, trauma, or anoxia all stimulate inflammatory processes [224]. The data suggesting an (statistical) influence of season of birth [116] is also consistent with the hypothesis as infectious epidemics often follow seasonal patterns. Some of the minor physical anomalies such as unusual scalp hair patterns and dermatoglyphic changes are explained because the development of these phenomena are linked to each other [225], to the development of the central nervous system [226], and are developmentally modulated by the pleiotropic effects of the same substances that modulate brain vascular development (e.g., vascular endothelial growth factor/vascular permeability factor [227] and epidermal growth factor [228]). The waxing and waning of symptoms would correspond to waxing and waning of inflammations as individuals are exposed, recover, and then re-exposed in conjunction with other physiological and hormonal influences, as seen in lupus [229]. The nature and severity of symptoms would depend on where in the brain the inflammation takes place and this may be stochastic. As the micro- vascular system is everywhere in the brain, lesions could produce the variety of symptoms seen in schizophrenia including dysfunctions of thought, emotion, memory, motor skills and autonomic regulation. The developmental age of the individual will also make a difference. Inflammatory processes that alter angiogenesis during prenatal development will likely have more dire consequences than inflammatory reactions that start after CNS maturation although even the adult brain remains susceptible [230]. We have attempted to schematically illustrate this dynamic process in Figure 2.

This theory also captures many of the little oddities observed in schizophrenia. For example, the reported abnormalities of the nail fold capillary beds seen in some people with schizophrenia [44] are also seen in people with inflammatory disorders such as FMF [231] and rheumatoid arthritis [232]. Another oddity is the negative association between schizophrenia and rheumatoid arthritis [45]. There are parallels in the post-streptococcal syndromes where RF and acute post-streptococcal glomerulonephritis very rarely occur in the same patient [233]. Some strains of group-A-streptococci identified by their M-protein serotypes are rheumatogenic while others are nephritogenic [233,234]. Phage or phage-like elements inserted into the streptococcal DNA are a major source of variation between streptococcal strains and these elements determine pathogenicity [235]. Additionally, host variation in humoral and cellular immune response shape the outcome of infection[211] By analogy, individuals with vascular/CNS involvement following, for example, streptococcal infections may be systematically spared from joint involvement as a function of both the invading strain and the individuals susceptibilities. Alternatively, as postulated for Alzheimer disease (cited earlier) that is also less common in people treated for arthritis, the anti-inflammatory treatments for arthritis might reduce the risk of inflammatory brain disease.

Another line of evidence compatible with this theory is the observation that genetic linkages for schizophrenia coincide with sites for glial growth factor cell regulators [214] and, as we have seen, the glia are key intermediaries of CNS inflammation and vascular regulation. More specifically, emerging data demonstrate associations between schizophrenia and genetic polymorphisms in regulators of inflammation such as tumor necrosis factor alpha genes [236,237] and interleukin-1 genes [238]. Another piece that fits into the puzzle is the fact that neuroleptics have inflammatory modulating properties [239-244] and neuroleptic treatment may be synergized by addition of anti-inflammatory drugs [245].

It may well be that the environmental components of psychiatric illness such as schizophrenia are relatively minor, ubiquitous, or chance events [246,247] that have the potential to stimulate the inflammatory systems. However, the nature of the insults may be less important than individuals' genetically influenced and idiosyncratic responses to the insults, similar to individuals with FMF who have an exaggerated inflammatory response. Thus, the genetic components of the inherited predisposition to mental illness may lie "upstream" in the immune system rather than in the CNS per se. The possibility that the environmental agents may be nearly universal (e.g. who has not had a strep throat or viral syndrome?), will mean that the prevalence of the etiological factor will be similar in control and experimental groups thus making it too easy to dismiss key environmental factors in null hypothesis designs [47,248]. Rather than focus on the environmental contributors that could be non-specific and ubiquitous, it will be more productive to look for genotypes that respond abnormally to triggers of inflammation and microvascular dysfunction (cf[48]). These individuals would be the ones who are at high risk for psychiatric illness. However, the inflammatory processes involve a cascade of steps involving many genes. But this, too, fits with the polygenic features of schizophrenia [249]. Identification of high-risk individuals, combined with such tools as immunizations or anti-inflammatory agents may promote prevention of much psychiatric morbidity. Already, the cytokine regulator and vascular growth factor erythropoietin is suggested as a possible neuroprotective factor in schizophrenia [250]

### Future directions

The speculations about psychoses developing from vascular/inflammatory processes provide direction for future research across many domains. In addition to pursuing direct evidence of altered activities in inflammatory/immune systems in people with psychoses, the inflammatory/vascular theory has implications for epidemiology, genetics, neuroimaging and neuropathology. For the epidemiologist, the challenge will be to detect relatively small signals against a very noisy background. We hypothesize that the triggers for inflammation can be many and varied and are common factors in the environment. Imagine starting with the clinical syndrome of Sydenham chorea and comparing the rates of strep throat in those affected vs. comparison sample of people without Sydenham chorea. Null hypothesis testing with small sample sizes and nearly ubiquitous etiological agents are clearly not adequate. A second epidemiological challenge is to cast a broad enough net to capture the wide variety of possible contributing factors. Rather than taking a one by one approach to exploring the etiological contributions of, say, virus titers, anoxia, physical trauma, the epidemiologist should look for any and all. It would be predicted that individuals with multiple "hits" (e.g. in utero exposure to virus and low Apgar scores and childhood head trauma) would be at greater risk than those exposed to just one event. If in utero inflammatory processes are active in the genesis of schizophrenia we would also predict an increased rate of fetal deaths in families of schizophrenic probands. A third epidemiological opportunity lies in the search for non-psychiatric inflammatory-related disease or traits in people with psychosis. If something is askew in the inflammatory process in schizophrenia, the effects will show up in other parts of the body. Though requiring replication, the association of psychosis with hemolytic anemia in lupus [251] provides an illustrative example. In addition to rheumatoid arthritis, the associations of diabetes and cancer have been explored in schizophrenia; one of is exploring rheumatic heart disease [205]. Population-based health registries should be used in a search for co-morbid physical illness.

For geneticists, the proposed theory obviously points to linkage/association studies using inflammation genes; a few examples were cited previously [236-238]. A simple step with extant data might start with a meta analysis defining chromosomal "hot spots" for linkage with schizophrenia and search the gnome maps for immune regulators at these sites as Moises, et al [214] have done for glial growth regulators. Family, twin, and adoption methodologies can all be applied to the issue of co-morbid or co-segregating physical conditions.

The inflammatory/vascular theory has much to suggest to neuroimaging research especially in the realm of reinterpreting regional perturbations in metabolic activity as primary disturbances of flow regulation rather than intrinsic neuronal metabolic abnormalities. It would be interesting to assess the impact of vasoactive compounds and inflammatory modulators on neuroimaging studies of regional blood flow. Likewise, further pursuit of neuroimaging evidence of disrupted blood brain barrier, as initiated by Dysken, et al [252], and with manipulation of inflammatory systems as suggested by Mueller and Ackenheil [253] would test our hypothesis.

The neuropathology of schizophrenia, focused mostly on the neurons, is notable for inconsistencies in findings (see [51,254] for reviews). Such inconsistency is exactly what would be predicted by an inflammatory/vascular theory where the lesions are truly functional in the sense that the function of the brain alters in relation to perturbations in blood flow regulation. Only the more prolonged and serious inflammation will leave visible traces of neuronal damage and such damage may be patchy and inconsistent from one patient to another. However, over the early years of CNS development, alterations in cellular organization or migration may result from disrupted angiogensis that must go hand in hand with neuronal and glial development. The location and extent of CNS change will be a function of severity of inflammation and timing during development. Such consequences will be hard to demonstrate in human post-mortem tissues and animal or in vitro models may be more fruitful areas for study the effects of inflammation on neurogenesis and blood flow regulation. To our knowledge, human post mortem studies have not utilized vascular cast methodology and this should be considered, perhaps casting one half of a specimen brain while subjecting the other half to cellular analysis.

### Specificity

Because of our interests and expertise, we have focused our attention on schizophrenia as the behavioral phenotype resulting from inflammatory-vascular pathology but the theory presented here is likely to be more general. Indeed, our use of examples of psychoses associated with known inflammatory- vascular pathologies (e.g. autoimmune CNS vascular disease or infectious CNS vascular disease as seen in syphilis) makes it clear that a vascular-inflammatory theory may apply to a wide range of psychotic conditions that may also include psychoses associated with mood disorders. Whereas, the classical genetic studies support the separateness of schizophrenia and mood disorders [255], there are modern molecular signs that schizophrenia and mood disorders share genetic elements in common [256,257]. Furthermore, mood disorders, like schizophrenia, show evidence of frontal lobe pathology, enlarged ventricles, abnormal cerebral blood flow [33,258] and vascular abnormalities [124]. To what extent all of these changes are epiphenomena of being psychotic (treatment effects or stress, etc) remain debatable [259].

However, finding similar brain changes in a variety of psychotic conditions does not necessarily mean these changes are epiphenomena. Examples from neuropsychiatry teach us that the underlying pathology does not necessarily define the behavioral symptoms. Thus, psychoses with Huntington disease may be affective-like or schizophreniform. Similar pathophysiological mechanisms may underlie a variety of psychotic phenotypes. The evolution of behavioral symptoms for any given pathophysiology may depend on a variety of moderating variables such as an individual's developmental age when the disease process begins, gender, hormones, genetic 'landscape' upon which the disease process unfolds, along with the nature, frequency, and intensity of successive triggers of inflammatory response.

### Reprise

A broad spectrum of observations leads to a working hypothesis that schizophrenia and, possibly, other psychiatric syndromes are the result of genetically mediated inflammatory reactions that damage the neuron-glial-capillary triad with resultant loss of ability to fine tune regional brain metabolism. This hypothesis incorporates genetic, epigenetic [260], and environmental factors. Furthermore, an inflammatory/vascular theory can explain the variety of behavioral symptoms seen in schizophrenia, the variable course of the illness, and the numerous other puzzling observations such as an excess of minor physical anomalies. Should this theory prove heuristic, it would point to the use of inflammatory modulators in treating the illness. Perhaps more importantly, identifying individuals who were at high risk for the disorder in high genetic risk families as well as the general population, because of abnormalities of their inflammatory systems, holds hope for prevention through early intervention using inflammatory modulators.

## List of abbreviations

ADHD attention deficit hyperactivity disorder

BDNF brain derived neurotropic factor

CBF cerebral blood flow

CNS central nervous system

DZ dizygotic

FMF familial Mediterranean fever

MZ monozygotic

NGF nerve growth factor

NO nitric oxide

PANDAS pediatric autoimmune neurological disorder associated with strep.

RF rheumatic fever

RHD rheumatic heart disease

VEGF vascular endothelial growth factor

## Competing interests

The author(s) declare that they have no competing interests.

## Authors' contributions

This article was the joint effort of both authors with input as noted below.

## Pre-publication history

The pre-publication history for this paper can be accessed here:
